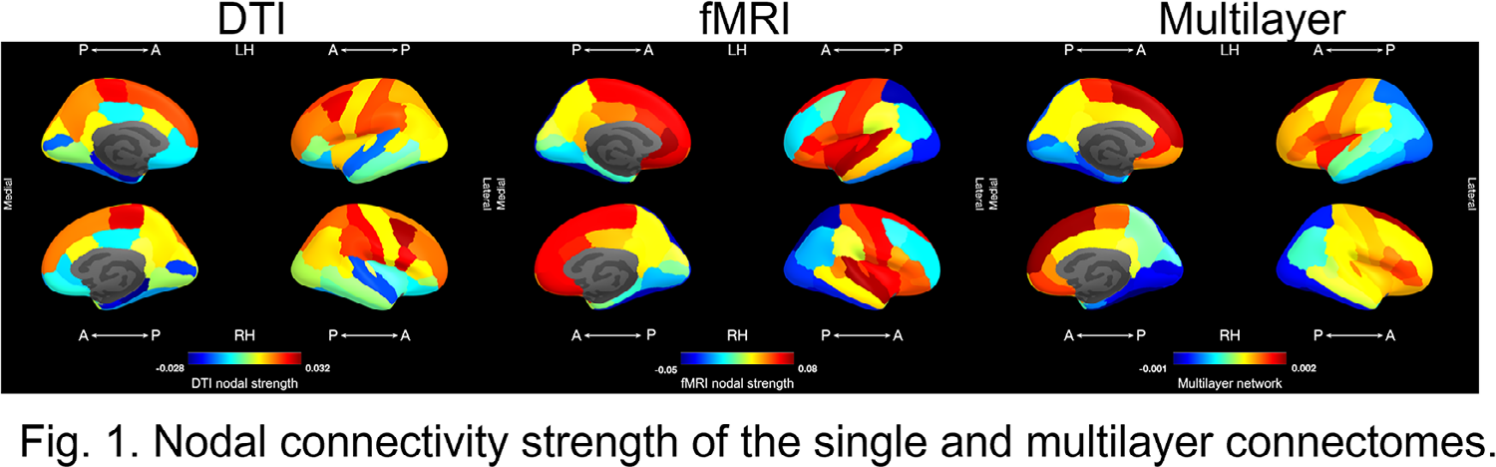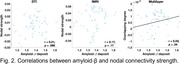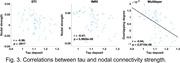# Multilayer structural‐functional connectome changes are associated with amyloid‐β and tau pathologies in Alzheimer’s Disease

**DOI:** 10.1002/alz.091424

**Published:** 2025-01-09

**Authors:** Meichen Yu, Olaf Sporns, Andrew J. Saykin

**Affiliations:** ^1^ Indiana Alzheimer's Disease Research Center, Indiana University School of Medicine, Indianapolis, IN USA; ^2^ Department of Psychological and Brain Sciences, Indiana University, Bloomington, IN USA; ^3^ Indiana Alzheimer's Disease Research Center, Indianapolis, IN USA

## Abstract

**Background:**

Brain network studies in Alzheimer’s disease (AD) have primarily focused on structural and functional connectomes as separate entities. However, it remains unclear how brain structure interacts with brain function in AD.

**Method:**

We included 75 cognitively unimpaired participants and 49 patients with AD. For each participant, we integrated a structural connectome (derived from DTI) and a functional connectome (derived from fMRI) into a multilayer structural‐functional connectome. We partitioned the DTI and fMRI maps into 68 Desikan‐Killiany ROIs. The links within the DTI and fMRI layers were formed by the structural and functional connectivity between the 68 ROIs, respectively. The interlayer links were defined by Pearson’s correlations between the connectivity vectors of ROIs from the two layers. We computed centrality values for each layer (i.e., nodal strength) and across layers (i.e., multiplex overlapping degree) and compared the centralities between patients and controls. We assessed spatial correlations between regional levels of amyloid‐β and tau pathologies and regional centrality values.

**Result:**

In general, ROIs that showed higher centralities in both layers also showed higher multilayer centralities, while ROIs with both a low structural and functional connectivity were typically observed in the multilayer network. Multilayer centrality was lower in AD patients relative to controls at multiple brain areas, such as the entorhinal cortex, superior frontal, temporal, and parietal cortices. Both multilayer and single‐layer centralities showed stronger correlations with tau than with amyloid‐β loads. Of note, only the multilayer centralities were positively correlated (r = 0.23; p = .04) with the amyloid‐β levels, although DTI‐derived centralities showed a trend‐level correlation (r = 0.21; p = .086) with amyloid‐β deposit. In addition, all the three types of centralities showed significantly negative correlations with tau deposit levels. Of note, multilayer centralities showed a stronger correlation than single‐layer metrics, in the order of multilayer (r = ‐0.54; p < .0001) > fMRI (r = ‐0.47; p < .0001) > DTI (r = ‐0.38; p = .0017).

**Conclusion:**

Our findings suggest that network centralities derived from a multilayer connectome might be more sensitive than those generated from each single layer in tracking and predicting amyloid‐β and tau spread.